# Osimertinib and Ramucirumab Induced Pyogenic Granulomas: A Possible Synergistic Effect of Dual Oncologic Therapy

**DOI:** 10.7759/cureus.15076

**Published:** 2021-05-17

**Authors:** Robert P Daze, Jewell Dinkins, Matthew H Mahoney

**Affiliations:** 1 Dermatology, Largo Medical Center, Largo, USA; 2 Dermatology, Meharry Medical College, Nashville, USA; 3 Dermatology, Mahoney Dermatology, Pinellas Park, USA

**Keywords:** pyogenic granuloma, egfr, vegf, osimertinib, ramucirumab

## Abstract

Pyogenic granulomas represent benign vascular tumors that can present on the skin and mucous membranes. Multiple etiologic agents have been implicated in the pathogenesis including several systemic medications. Two notable oncologic therapies, epidermal growth factor receptor inhibitors and vascular endothelial growth factor receptor inhibitors, have each been associated with drug-induced pyogenic granulomas. We report a novel case report of dual therapy, medication-induced pyogenic granulomas. This likely represents a synergistic relationship between an epidermal growth factor receptor inhibitor, osimertinib, and a vascular endothelial growth factor receptor inhibitor, ramucirumab.

## Introduction

Pyogenic granulomas, or lobular capillary hemangiomas, are benign vascular proliferations of the skin and mucous membranes. Grossly, these lesions present as polypoid papules and nodules with a propensity to ulcerate. Although multiple causative agents have been implicated in the pathogenesis, various systemic medications are known triggers for this vascular anomaly [1,2]. Notably, pyogenic granulomas occur in 10% to 30% of cases where epidermal growth factor receptor inhibitors are used for the treatment of advanced malignant tumors [1]. More recently identified in the literature, vascular endothelial growth factor receptor inhibitors present as a paradoxical etiology in the development of pyogenic granulomas. Our clinical observation identified a novel and likely synergistic interaction between an epidermal growth factor receptor inhibitor, osimertinib, and a vascular endothelial growth factor receptor inhibitor, ramucirumab.

## Case presentation

**Figure 1:**
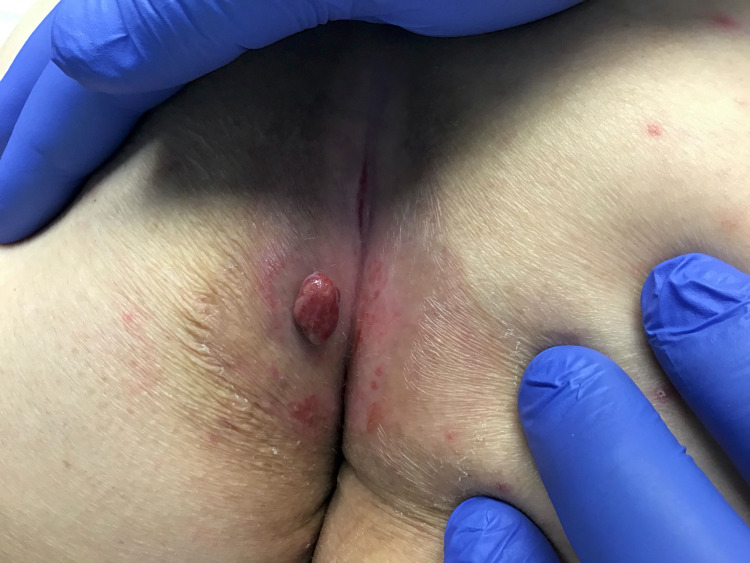
Solitary, friable, red polypoid nodule present on the left medial buttock.

**Figure 2:**
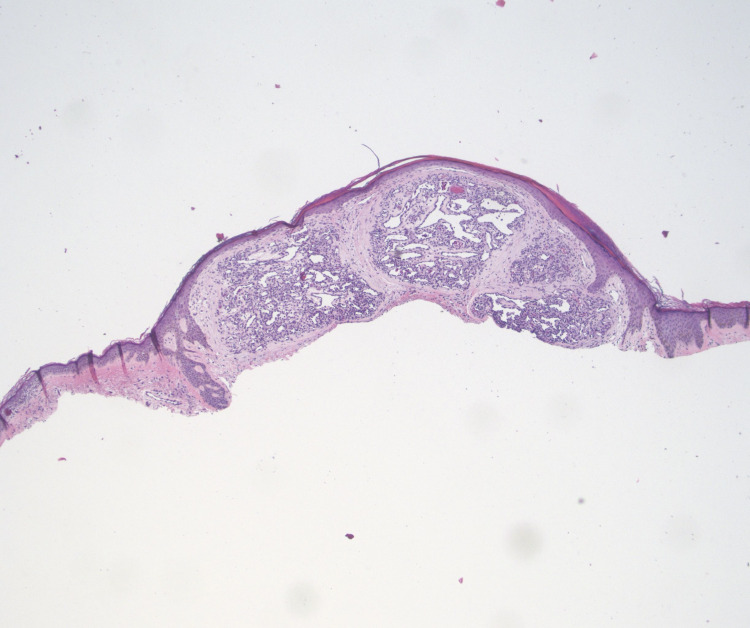
Shave biopsy demonstrating a capillary proliferation in a multi-lobulated pattern encased by an epithelial collarette, supporting a diagnosis of a pyogenic granuloma (low power 40x, hematoxylin-eosin stain).

**Figure 3:**
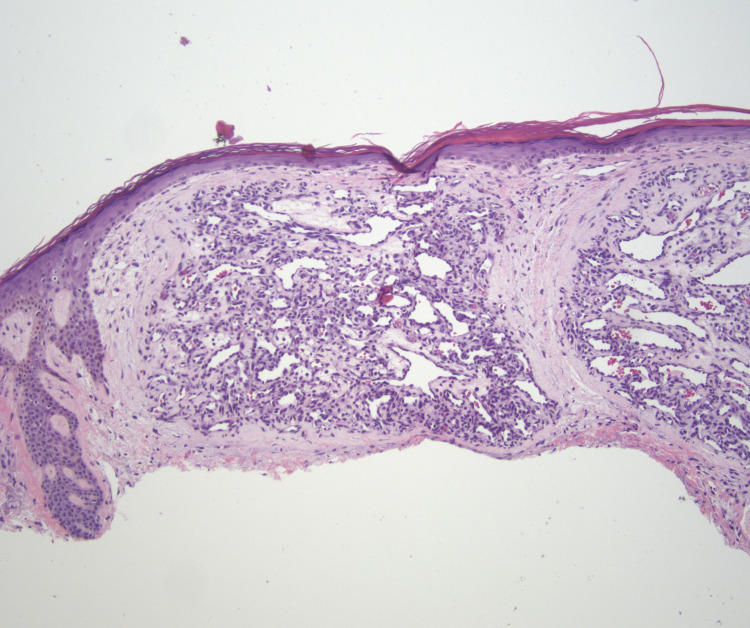
Shave biopsy demonstrating a capillary proliferation in a multi-lobulated pattern encased by an epithelial collarette, supporting a diagnosis of a pyogenic granuloma (medium power 100x, hematoxylin-eosin stain).

A 68-year-old Caucasian female with a history of stage IV non-small cell lung cancer and notable epidermal growth factor receptor exon 19 mutations presented with a new tender bright red nodule present on the left buttock, with onset approximately three months prior to presentation. The patient endorsed rapid growth and intermittent bleeding but denied any previous trauma to the area. The patient had a similar lesion present on her right upper chest. Since her lung cancer diagnosis in April 2020, the patient has been successfully treated with combination therapy of osimertinib, a third-generation epidermal growth factor receptor inhibitor, and ramucirumab, a vascular endothelial growth factor receptor inhibitor. Physical examination demonstrated a solitary, friable, red exophytic nodule located on the left superomedial buttock at the gluteal cleft and measuring 1.6 x 1.0 x 0.7 cm (Figure 1). A similar lesion was present on the right upper chest measuring 0.6 x 0.5 cm. Shave biopsies were obtained from both sites and were evaluated by hematoxylin-eosin staining. Each specimen demonstrated an exophytic lesion with a proliferation of small capillaries in a lobular pattern separated by fibrous septae. These histopathological findings in both shave biopsies were consistent with pyogenic granulomas (Figures 2, 3). After shave removal the area was treated with a curettage and electrofulguration, with no subsequent clinical recurrence. Given the clinicopathologic setting, we believe these tumors very likely occurred secondary to the patient’s current oncologic therapy; both medications are known etiologic agents in the formation of pyogenic granulomas.

## Discussion

Pyogenic granulomas, also known as lobular capillary hemangiomas, are benign vascular tumors that can affect the skin and mucosal membranes. Multiple causative agents have been implicated in the pathogenesis including chronic irritation, prior trauma, hormones, and systemic medications such as retinoids, indinavir, and epidermal growth factor receptor inhibitors [2]. We report a case of eruptive pyogenic granulomas as a proposed synergistic effect of two systemic medications known to cause these vascular tumors: epidermal growth factor receptor inhibitors and vascular endothelial growth factor receptor inhibitors [3,4]. To our knowledge this is the first documented case in the literature highlighting the combined synergy between these two oncologic medications.

Epidermal growth factor receptors affect cellular differentiation and proliferation. Dysregulation or overexpression of this receptor can lead to tumor-induced angiogenesis [5]. Osimertinib is a third-generation kinase inhibitor of the epidermal growth factor receptors. It binds irreversibly to certain mutant forms of epidermal growth factor receptors likely minimizing the cutaneous and gastrointestinal adverse events of its predecessors. This selective inhibition likely diminishes but does not fully negate the potential for adverse toxicities [2]. As a medication class, epidermal growth factor receptor inhibitors are associated with a wide spectrum of dermatologic toxicities including an early onset papulopustular or acneiform eruption, xerosis, pruritus, and photosensitivity [5]. Epidermal growth factor receptor inhibitor therapy is also associated with paronychia, which may lead to the development of pyogenic granuloma like lesions [5,6]. These lesions typically occur 4-8 weeks after treatment but can be a late manifestation seen up to six months later as seen in our patient.

Ramucirumab is a fully human immunoglobulin G1 (IgG1) monoclonal antibody that targets the extracellular domain of vascular endothelial growth factor receptor 2, blocking the binding site for various vascular endothelial growth factor-related ligands. The most common adverse events with the systemic infusion are non-dermatologic including hypertension, peripheral edema, hypoalbuminemia, thrombocytopenia, and fatigue [3,7]. Vascular tumors are not a well-known complication of vascular endothelial growth factor therapy as angiogenesis is suppressed. In the phase I clinical trial of CDP791, a vascular endothelial growth factor receptor-2 inhibitor, seven out of 31 patients developed benign hemangiomas after three cycles of therapy [8]. Lim et al. described the growth of a vascular tumor, resembling a tufted angioma, during ramucirumab treatment [7]. This particular tumor harbored a single somatic mutation in the knockdown resistance gene which encodes for vascular endothelial growth factor-2, likely conferring an aberrant mechanism to vascular tumorigenesis. Testing for this particular mutation was not performed in our case. This paradoxical angiogenic event during ramucirumab therapy was noted in subsequent case reports, albeit with other combined oncologic therapies such as paclitaxel and docetaxel [4,7,9,10]. The timing of the tumor onset ranged from approximately two weeks to six months after administration with multiple sites affected including the mucosa, nail apparatus, trunk, and extremities [4,7,9,10]. While vascular endothelial growth factor inhibition is thought to downregulate the tumoral angiogenesis, the mechanism behind this paradoxical reaction remains largely undefined.

## Conclusions

In conclusion, we report a case of pyogenic granulomas arising in the setting of epidermal growth factor receptor and vascular endothelial growth factor receptor inhibition. While other combined therapies have been documented in the literature, to our knowledge this is the first case of pyogenic granulomas arising during this specific concomitant therapy. While individually each medication is implicated as a causative agent, we propose this combined therapy to be a manifestation of a synergistic effect on vascular endothelial cell proliferation. While further studies are necessary to delineate this mechanistic interaction, it behooves physicians to recognize this novel adverse event and monitor for the development of vascular tumors.
